# Empirical dynamics of railway delay propagation identified during the large-scale Rastatt disruption

**DOI:** 10.1038/s41598-020-75538-z

**Published:** 2020-10-29

**Authors:** Beda Büchel, Thomas Spanninger, Francesco Corman

**Affiliations:** Institute for Transport Planning and Systems, ETH Zürich, 8093 Zürich, Switzerland

**Keywords:** Civil engineering, Complex networks

## Abstract

Transport networks are becoming increasingly large and interconnected. This interconnectivity is a key enabler of accessibility; on the other hand, it results in vulnerability, i.e. reduced performance, in case any specific part is subject to disruptions. We analyse how railway systems are vulnerable to delay, and how delays propagate in railway networks, studying real-life delay propagation phenomena on empirical data, determining real-life impact and delay propagation for the uncommon case of railway disruptions. We take a unique approach by looking at the same system, in two different operating conditions, to disentangle processes and dynamics that are normally present and co-occurring in railway operations. We exploit the unique chance to observe a systematic change in railway operations conditions, without a correspondent system change of infrastructure or timetable, coming from the occurrence of the large-scale disruption at Rastatt, Germany, in 2017. We define new statistical methods able to detect weak signals in the noisy dataset of recorded punctuality for passenger traffic in Switzerland, in the disrupted and undisrupted state, along a period of 1 year. We determine how delay propagation changed, and quantify the heterogeneous, large-scale cascading effects of the Rastatt disruption towards the Swiss network, hundreds of kilometers away. Operational measures of transport performance (i.e. punctuality and delays), while globally being very decreased, had a statistically relevant positive increase (though very geographically heterogeneous) on the Swiss passenger traffic during the disruption period. We identify two factors for this: (1) the reduced delay propagation at an international scale; and (2) to a minor extent, rerouted railway freight traffic; which show to combine linearly in the observed outcomes.

## Introduction

The interconnectivity of transport networks makes them vulnerable when parts of the network experience a reduced performance, or fail^1^. A performance reduction caused by smaller or larger disruptive events has specific geographic and temporal dynamics of reduced transport performance^2^. Typically, disruptive events triggered in a specific limited area result in a degradation of performance, which then propagates in the network. The system stabilizes at a less performing state, also considering some management actions, and finally recovers with a certain rapidity towards its original performance.

In railway networks, non-performance results in delays, which propagate through the system. The mechanisms of delay propagation directly reflect the complex, concurrent and co-occurring processes characteristics of railway operations. To disentangle them, we study delay propagation (i.e. how railway systems are vulnerable to small reduced performance), through comparison of the same railway system in two operating conditions. We exploit a unique chance in the real world, to observe a systematic change in railway operations based on empirical data, without the typically co-occurring changes in infrastructure or timetable, given by the large-scale railway disruption at Rastatt, Germany, in 2017. The quantification of delay propagation clarifies how the disruption had a measurable effect on the Swiss network.

The quantification of the vulnerability of transport networks has been attracting much interest^3–5^. In topological studies of link or node failures, network-based properties are predominant^6–9^. Including service level aspects allows quantifying travel time and capacity^10,11^. Determining the relation between link, node, network characteristics, and risk or exposure of a theoretical small or large disruption, allows a-priori quantification of resilience, and determination of strategic actions such as reducing vulnerability of some links^8,9,12–14^. Once a disruption happened, management and recovery actions (changing network structure and usage of links and nodes) can be quickly computed and disseminated to users^2,6,15–19^. The costs of all those actions and their consequences can be estimated^20–24^, together with aggregate measures of resilience and rapidity of recovery. Only few works^3^ study empirical dynamics of existing disruptions in their effects to transport performance, or choices of travelers^20–22,25–27^, possibly due to unavailability of data, or complexity of interacting effects. With the recent outbreak of COVID-19 and restricted mobility, a large interest in studies on demand or supply changes developed^28,29^.

Different modes of transportation are affected by non-performance in different ways, due to the specific characteristics of links and nodes in terms of technology, speed, capacity, and limitations imposed by a disruption. The changes in performance at the onset of, and during the disruption, depend on the specific transport mode and disruption. On most road and pedestrian networks, limitations occur exclusively at links or nodes (prohibiting passage, restricting speed or flow^30^). Routes (sequences of consecutive links) can be affected if any of their links is affected. Availability of vehicles (or ability to walk) is most often assumed to be sufficient, and links have continuous availability through time, as vehicles/persons can in general use them at any time. On air networks, limitations occur at nodes (reduced runway availability or capacity), and on links (events in the atmosphere such as unsuitable weather, or volcanic activity)^20,21,24^. To operate a network, vehicles and crew are needed, as well as the availability of an air corridor i.e. a time-space path where airplanes can move safely. Route failures are not particularly relevant as most air transport relations are point to point; but passenger transfers at hubs might propagate impacts network-wide. On maritime networks, the capacity of links (i.e. the oceanic course of a vessel, apart from bottlenecks such as canals or straits) is much larger than available capacity at hubs (ports and equipment for berthing and transshipment). The links themselves, i.e. the oceanic course of a vessel, are operated point-to-point, and rarely completely disrupted^27^. The availability of vehicles and crew is crucial in operating the network. In road-based public transport, such as buses, limitations occur at links or nodes (similar to cars, prohibiting passage)^10,11^. Vehicles, crew, and routes are required to operate services. Routing alternatives can avoid a disruption, but those are typically scarce as infrastructure is not available, or congested, and public transport services are bound to run at some planned times only. In those cases, the network exhibits continuous characteristics (vehicles can use links at any time), and discrete characteristics (few vehicles, specific time to use a node, fixed capacity of a node).

In railway-based transport, restrictions come from unavailable links or nodes; unavailable infrastructure access, i.e. a time-space path where trains can safely move; and unavailable vehicles or crew^31^. Trains are separated by a safety system ensuring a separation over the block sections of the infrastructure, and cannot overtake each other along the tracks. Infrastructure is typically scarcely available^32^, and rarely provides usable alternative routes between any two points, compared to roads, seaways or air corridors. Thus, the network exhibits many discrete characteristics due links and nodes, whose capacity is available only at the times planned in the timetable. As a result, the effects of disruptions are propagated along nodes and links, over the entire length of the infrastructure, at microscopic scope (signals, block sections)^19,33,34^, rather than at macroscopic scope (stations, networks). This latter phenomenon is characteristic of railway networks, as even in undisrupted situations, smaller disturbances propagate and spread throughout the network^32,35,36^. It is in fact the safety system, which slows down and stops trains in front of signals, increasing their delays, when they are running too closely. Overall, both small and large disruptions result in delays, i.e. deviations between the planned timetable and actual operations. Delays can only be measured where a planned timetable is given, most often, only at stations with a planned stop. Concluding, railway networks differ greatly from other transport networks due to interacting discrete and continuous dynamics, at microscopic and macroscopic scale, and the complex mechanism of delay propagation mediated by infrastructure and timetable. This results in a different response pattern from the other (continuous) modes. The delay propagation phenomenon is known to be stronger in geographical (area affected^31^), temporal aspects (time to return to normality^33^) and intensity (delay actually experienced) in very utilized networks (amount of trains running per amount of time over a given microscopic infrastructure resource). Approximate representations of this effect at system level, with limited microscopic detail, hint at some exponential scaling^8,35,36^ governed by few parameters. In practice, this relation is very complex and based on a large set of inputs. Delay propagation models estimate the expected performance of a system, based on a plan of operations and assumed initial delays. A precise estimation of delay propagation allows designing appropriate buffer times in the timetable, able to reduce delays and their propagation (similar to a non-linear damping process). Simpler approaches use deterministic approximate relations based on the number of trains running, and buffer times^32^, assuming full availability of infrastructure, vehicles, and drivers. Stochastic approaches separate factors dependent and independent from the topology^36^; or exploit Bayesian frameworks^37^, Monte-Carlo approaches^38,39^, or simulation^40^.

The extent by which current railway delay propagation models match the observed empirical dynamics under co-occurring disturbances and interrelated processes, mediated by infrastructure availability and planned timetable, is not clear^31,37^. The calibration and validation of models would require a systematic change in railway operations, without any change in infrastructure or timetable (i.e. controlled experiment), which is very rare in reality. Moreover, railway disruptions have been subject to limited study^3,5,22,41^ probably due to exactly those complex characteristics of delay propagation, and their interplay with management actions aiming to keep the system running^31^, often taken under strong time pressure and resulting suboptimal in hindsight^17,19,42^.

**Figure 1:**
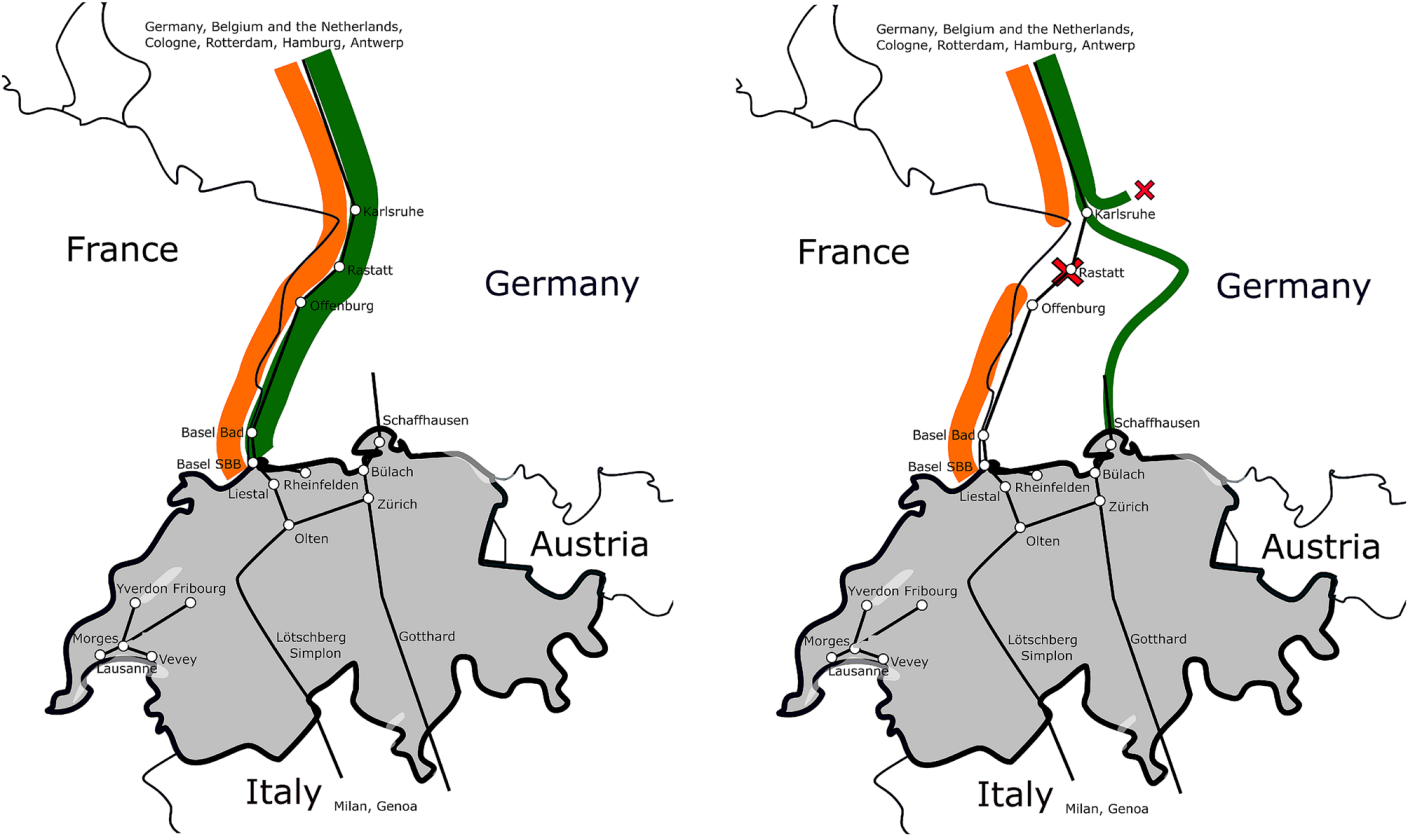
Graphical representation of Switzerland, boundary points considered, disruption location and schematic traffic entering Switzerland from Germany (left: undisrupted case; right: disrupted case. Orange: passenger traffic, roughly 200 trains per week per direction, in both disrupted/undisrupted case; green: freight traffic, roughly 250 trains per week per direction, in the undisrupted case, up to one quarter of which is canceled or not entering Switzerland in the disrupted case). Own elaboration from a public domain source https://commons.wikimedia.org/wiki/File:Blank_political_map_Europe_in_2006_WF.svg.

We fill this gap in the literature of railway delay propagation models, by studying the Swiss railway system in two uniquely different states, over a real-world experiment, on 1 year of empirically recorded data of passenger railway traffic. We exploit the occurrence of the major disruption at Rastatt, Germany, blocking the major railway European corridor for 2 months, to study how changes in observed railway delay propagation can be traced back to different, disentangled, mechanisms. We define multiple novel metrics and statistical methods to compare the disrupted and undisrupted state.

We distinguish (explicit) mechanisms that we can associate beyond a certain level of confidence to the disruption itself, from the many (implicit) variations, noises, and disturbances that happened irrelevant of the disruption, and with no precisely identifiable root cause. We determine how delay propagation changed, and quantify the effects of the Rastatt disruption towards delay propagation in the Swiss network, more than two hundred kilometers away.

We focus on the Swiss network, where Basel SBB (Basel in short) and Schaffhausen are the border stations where cross-border trains coming from Germany and Rastatt have to pass. The Rastatt disruption resulted in two explicit changes to the railway traffic in Switzerland, see Fig. 1. Some freight trains coming from Germany and the North, along the Rhine Alpine corridor, changed route from the boundary point at Basel to the boundary point at Schaffhausen; up to a quarter of the freight traffic has been canceled or rerouted, not entering anymore Switzerland^43^. This is similar to a pressure decreased at one place, and applied in reduced form to a different part of the system. The passenger trains coming from Germany and the North have been short turned, and were running via Basel between Switzerland and Offenburg (150 km away) rather than via Basel between Switzerland and Hamburg or Berlin (700 km away). In the Swiss network, the planned timetable and infrastructure usage of all passenger trains remained the same, with no significant cancellations or adjustments. Nevertheless, the short turning of the long-distance international trains resulted in smaller variability of actual operations, and less entrance delays when entering into the Swiss network at Basel, which hints at a reduced epidemic spreading of delays in a network^30,36^.

We empirically verify how the specific propagation dynamics and cascading effects are heterogeneous, with different development, spreading and fade out, in the disrupted and undisrupted state. The Rastatt disruption, with large global negative effects, lead to actual measurable improvements of performance for passenger traffic, on part of the Swiss railway network, and smaller performance decrease on other parts. A simulation represents well the observed behavior, and shades light on the relative magnitude, and linear interaction, of the two explicitly modelled changes. In the specific test case, the propagation of a lower initial delay has a much larger network impact than a change and rerouting in freight traffic volume.

## Results

### Effects around Basel

We analyse with a set of proposed metrics (see detailed description in the Methods section) the variations in delay propagation of the passenger traffic arriving in Basel SBB from Germany; and passenger traffic (regardless if they were coming from Germany, or originated in Switzerland) at their first stop from Basel SBB. We cannot consider delay changes for freight traffic, as its volume was strongly affected by the Rastatt disruption; and more generally, freight traffic does not stop at stations thus having a ill-defined delay; and despite its buffer times are typically large, its performance is often erratic. Passenger traffic instead experienced no significant cancellation in Switzerland, and its timetable remained unchanged throughout the disruption.

We graphically report the time series of the 20th, 40th, 60th, and 80th percentile values of the delays of all long-distance passenger trains arriving at Basel SBB from Germany. We consider a moving average over 7 days, over the course of the timetable years 2017 and 2018.

**Figure 2:**
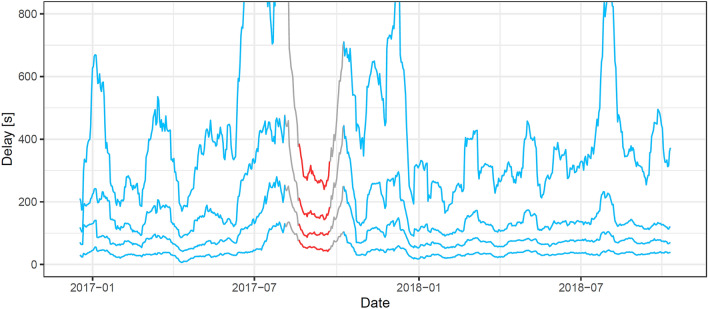
Recorded arrival delays (moving average of daily 20th, 40th, 60th, 80th percentile values) of trains arriving in Basel SBB from Germany, over timetable years 2017 and 2018. In blue, dates not influenced by the Rastatt disruption; in red, disrupted dates; in grey, the transition phase, where the moving average spans disrupted and undisrupted dates.

Throughout the 2 years, almost half of the traffic had more than 2 min of delay. The 20 and 40 percentile are more stable than the 60 and 80 percentile. The highest delays of this observation period of 2 years are reached just before and after the disruption, presumably due to the construction works in southern Germany. During the disruption, all percentile time series have distinctly lower delays than before and after the disruption period. The gap between the low and high percentile (i.e. the variation of the delays) is remarkably smaller during the disruption period. A reduced performance has been linked to a reduced variability of performance during a disruption also in other cases^5^.

**Table 1:** Metrics for the Basel area.

Indicator	*p*	Basel	Liestal	Rheinfelden	Olten	Zürich
d [min]	–	0	9	12	24	53
$I_{diff}$	0.8	0.711	0.971	0.841	0.883	0.984
	0.6	0.954	0.979	0.902	0.944	0.951
	0.4	0.936	0.977	0.936	0.823	0.835
	0.2	0.800	0.985	0.790	0.513	0.953
$I_{sum}$	0.8	0.631	0.966	0.849	0.749	0.975
	0.6	0.788	0.970	0.761	0.901	0.915
	0.4	0.799	0.961	0.864	0.582	0.883
	0.2	0.856	0.966	0.539	0.281	0.867
KS-test	0.8	$<10^{-09}$	0.012	1.8 $10^{-3}$	1.6 $10^{-3}$	0.010
	0.6	$<10^{-07}$	0.040	7.2 $10^{-4}$	7.0 $10^{-3}$	0.010
	0.4	$<10^{-06}$	0.019	$<10^{-05}$	0.038	0.014
	0.2	7.2 $10^{-3}$	6.3 $10^{-6}$	0.119	0.060	0.023
*t*-test	0.8	$<10^{-24}$	$<10^{-06}$	2.6 $10^{-3}$	$<10^{-06}$	$<10^{-09}$
	0.6	$<10^{-25}$	$<10^{-05}$	3.7 $10^{-2}$	$<10^{-06}$	$<10^{-06}$
	0.4	$<10^{-21}$	1.7 $10^{-3}$	$<10^{-06}$	1.8 $10^{-3}$	$<10^{-05}$
	0.2	$<10^{-16}$	9.4 $10^{-4}$	0.626	0.035	$<10^{-06}$
MQD	0.8	− 376.17	− 22.92	− 13.85	− 28.86	− 79.78
	0.6	− 150.05	− 13.49	− 9.30	− 21.33	− 36.82
	0.4	− 65.14	− 8.67	− 5.08	− 12.35	− 26.80
	0.2	− 18.98	− 6.45	− 6.32	− 7.90	− 21.38

Table 1 reports the quantitative evaluation using the novel metrics proposed, described in the Methods section, over the timetable year 2017. $I_{diff}$ quantifies the likelihood that the **single shocks** at beginning and end of the disruption occur elsewhere throughout the data (the higher, the more exceptional the observed behavior was during the disruption). $I_{sum}$ quantifies the likelihood that the **combined shocks** at beginning, and recovery at the end of the disruption occur elsewhere throughout the data (the higher, the more exceptional the observed behavior was during disruption). Both indicators report that the change during this period is rare (half of $I_{diff}$ and $I_{sum}$ are higher than 0.9), especially those for the highest percentile levels.

The KS-test reports the **statistical significance of the pattern observed in a 2 months horizon**, i.e. quantifying the likelihood that the samples observed within the disruption belong to the same distribution than before and after the disruption (the lower, the more exceptional the observed behavior was during the disruption). The *t*-test reports the **statistical significance of the pattern observed over the entire year**, i.e. quantifying the likelihood that the samples observed during the disruption have the same mean as the data observed outside of the disruption, over a period of 1 year (the lower, the more exceptional the observed behavior was during the disruption). Both statistical tests report the significance of the variation, with extreme strength in Basel and Zürich, and smaller strength in Rheinfelden and Olten, probably due to the different timetable structure and services running. At the 0.6 and 0.8 percentile, except Liestal, both KS-test and *t*-test metrics reported significance.

The Mean Quantile Deviation (MQD) gives the **magnitude of the variation** observed, in seconds. All reported values are negative, i.e. the delay decreased during the disruption, with the higher percentiles showing a larger decrease. In Basel, the decrease of the 80th percentile is more than 6 min, and 60% of the traffic had its delay reduced by a minute or more. In Zürich, the effect is a reduction of delay by around 30 s, up to almost 80 s for the highest percentile; the strongly delayed trains performed better in the disruption period. This signal is weak compared to the noise, i.e the median delay has been ranging between 120 and 300 s throughout year 2017. The performance change was not uniform, but depends on the percentile level, i.e. the prevailing delay.

### Effects around Schaffhausen

**Figure 3:**
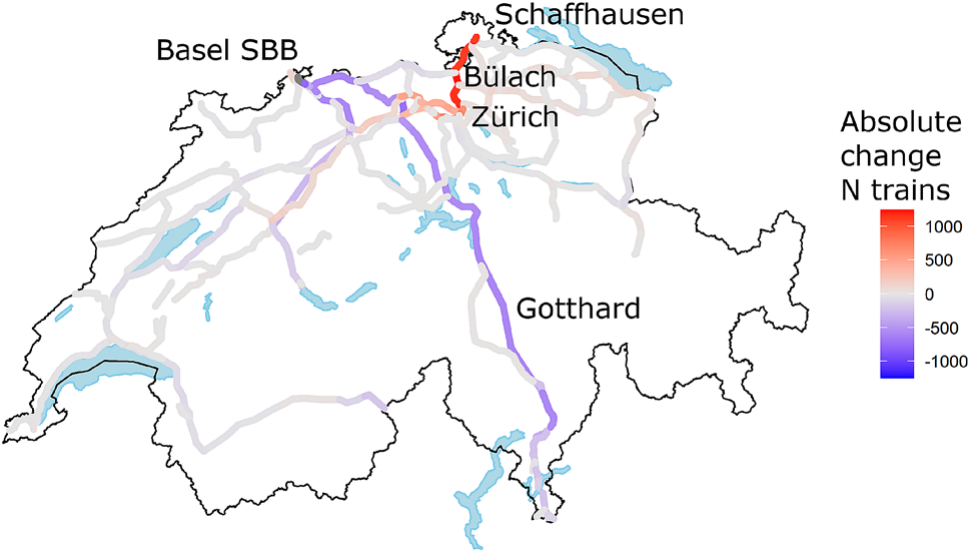
Observed change in monthly volume of freight train traffic during the disrupted period. Figure designed with R v3.6.3 https://cran.r-project.org:, package ggswissmaps v0.1.1 https://cran.r-project.org/web/packages/ggswissmaps/.

Due to the disruption, freight trains running on the Rhine Alpine corridor have been globally rerouted or cancelled. Figure 3 reports the absolute difference in number of freight trains actually running in the Swiss network, on an average month of the disrupted period compared with an average month in 2017. The different rail corridors have different total infrastructure capacity, and different ratio of passenger/freight trains. The increase in the Schaffhausen-Zürich corridor (red, 500 more freight trains per month, normally 500–1000 freight trains per month) is relatively much stronger than the decrease in the Basel–Gotthard corridor (violet, 1000 freight trains less per month, normally 1500–2500 freight trains per month).

The impacts of this variation in infrastructure utilization (by the freight traffic) on the performance of the Swiss passenger railway traffic (sharing the same infrastructure) are analysed with the proposed metrics in Table 2. We consider the passenger traffic arriving in Schaffhausen from Germany; and passenger traffic (regardless if they were coming from Germany, or originated in Switzerland) at their first stop from Schaffhausen. The number of passenger trains running (originating in Germany, or in Schaffhausen) remained unchanged throughout the disruption.

In Schaffhausen, the metrics report small and insignificant (*t*-test above 0.5) changes in delay. All other stations report a positive deviation in delays (MQD). The metrics $I_{sum}$ reports a higher value, i.e. more significant variations, than $I_{diff}$, and this reflects the slower onset of the management actions in the area. Rerouting of freight trains from Germany via Schaffhausen needed some start-up time; in the first days immediately after the disruption, freight companies preferred to wait, and they had permissions and resources (drivers with permission to run over this part of the network, as well as locomotives and train paths) only some time later^44,45^.

The reversion to a normal state at the end of the disruption, as identified by the cumulative shocks considered by $I_{sum}$, was instead rather rapid, and identified as significant. In fact, after the disruption the operation changed immediately back to the Rhine Alpine corridor, which offered faster travel times and larger infrastructure capacity.

The freight corridor used by the additional freight trains avoids major stations and had smaller and less significant impact to Schaffhausen and Zürich, than to Bülach, where passenger and freight traffic must necessarily interact. In this last station, the effect is strongest with almost half of the traffic facing an additional delay by half a minute or more; both KS-test and *t*-test show significance, one or more orders of magnitude smaller than $\alpha$ = 0.01.

Overall, the delay increase identified is remarkably different from the delay reduction seen for the Basel area, hinting at a strong heterogeneity between the two areas. Within the Schaffhausen area alone, the influences are again heterogeneous, with strongest impacts through microscopic delay propagation along the corridor, as experienced in Bülach. The quantitative analysis is in good agreement with the observed actions.

**Table 2:** Metrics for the Schaffhausen area.

Indicator	*p*	Schaffhausen	Bülach	Zürich
d [min]	–	0	19	36
$I_{diff}$	0.8	0.790	0.527	0.339
	0.6	0.874	0.311	0.951
	0.4	0.808	0.635	0.850
	0.2	0.313	0.799	0.879
$I_{sum}$	0.8	0.539	0.991	0.267
	0.6	0.718	0.955	0.840
	0.4	0.764	0.969	0.787
	0.2	0.733	0.994	0.867
KS-test	0.8	0.119	$<10^{-05}$	0.083
	0.6	0.119	$<10^{-05}$	0.212
	0.4	0.096	8.1 $10^{-4}$	0.473
	0.2	0.119	2.6 $10^{-3}$	0.437
*t*-test	0.8	0.626	$<10^{-08}$	0.041
	0.6	0.849	$<10^{-07}$	0.517
	0.4	0.598	$<10^{-06}$	0.265
	0.2	0.609	$<10^{-05}$	0.249
MQD	0.8	− 26.36	+50.58	+30.41
	0.6	− 8.86	+27.23	+7.81
	0.4	− 7.60	+17.73	+5.73
	0.2	− 4.94	+12.52	+4.03

### Global validity of the effects

We also investigated if some systematic variations happened at the same time in the Swiss network (co-occurrence) but were unrelated to the Rastatt disruption (a weak causality analysis^24^). We performed the same analysis for a Swiss train station of comparable size and traffic, far enough away from the disruption, that according to general theories of disruption impact, should have low or even unquantifiable effects. The data (reported as supplementary material) shows no specific evidence or special effect in the disrupted period, with $I_{diff}$ and $I_{sum}$ mostly around 0.5. The *t*-test identifies some significant changes for half of the stations investigated, while the KS-test identifies only one significant observation. We trace this to a large variability of yearly operations, and the variability encountered during the disruption has been no different than otherwise throughout the year. The MQD has an overall erratic behavior, with a negligibly small increase, and some decrease at different stations. This shows also how a single metric cannot describe the complexity and noise in the data, and a variation with both fast and persistent dynamics.

Overall, no clear and generalized variation of performance is evident elsewhere in Switzerland during the Rastatt disruption. The stations near Basel and Schaffhausen experienced a variation during the disruption, which was not significantly experienced in the rest of the Swiss network, around Lausanne. This matches well the currently accepted theory of spreading of disturbances throughout networks, which identifies a maximum geographical dimension of the impact, from few boundary points.

### Identifying magnitude of the root causes by simulation

We now discuss how a state-of-the-art simulation model, OnTime, based on typical delay propagation theory^39^ (the Methods section describes its assumptions and functioning, and its calibration for the test case) can partially replicate the degree by which the delay performance of passenger trains improved in the area of Basel, and decreased in the area of Schaffhausen. Moreover, we aim to identify which root cause is responsible for which observed effects, i.e., disentangling the effects of co-occurring actions, business processes, and operations. We compute the delay of passenger trains in the baseline condition (for a typical day, immediately before the disruption) and for a typical day during the disruption, by a calibrated mesoscopic stochastic simulation in OnTime, considering all traffic (passenger and freight) running in the Swiss network.

**Figure 4:**
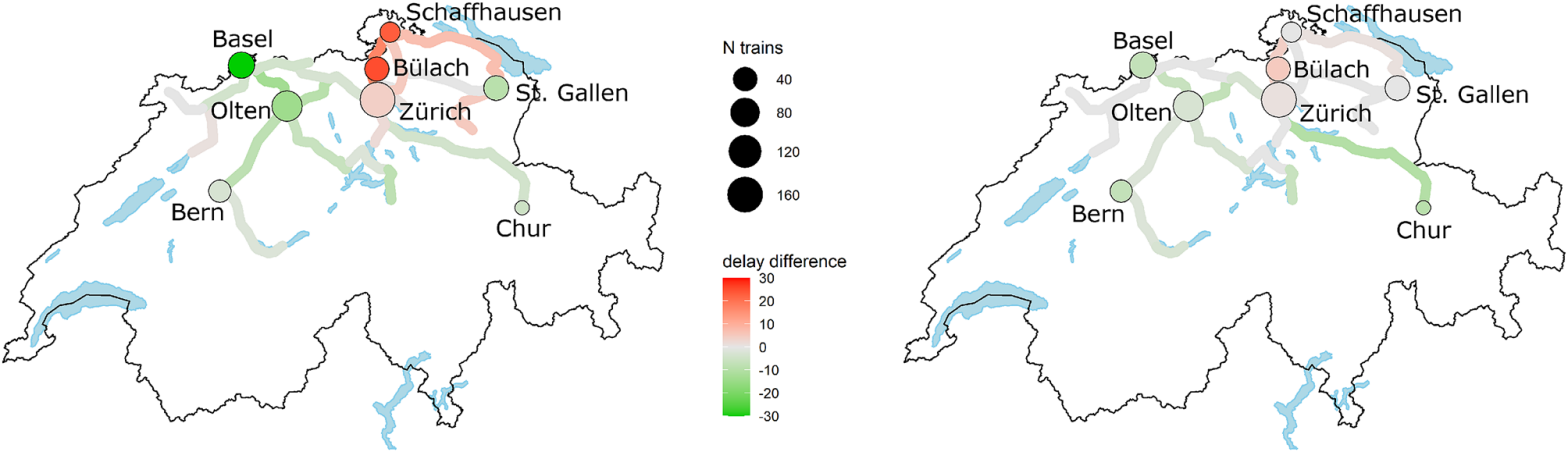
Graphical representation of effects across Switzerland. Left: observations; right: simulation. Figure designed with R v3.6.3 https://cran.r-project.org:, package ggswissmaps v0.1.1 https://cran.r-project.org/web/packages/ggswissmaps/.

Figure 4 reports the variation in median delay from undisrupted to disrupted situation (green-red color, mapped between $-30$ to $+30$ s for all individual stations and measurement points where trains pass and/or stop, green reports a delay decrease during a disruption) of all passenger traffic originated in the focal points at Basel or Schaffhausen, for the recorded data (left) and simulated traffic (right). Visually, a good agreement between the observed and simulated variation of delay propagation is present, especially when filtered by the volume of traffic (i.e. line Zürich-Chur). The variation along a line might be discontinuous, as the services running are heterogeneous, and have different stopping patterns; this stresses the need for a detailed study.

A set of 8 station areas is analysed more in detail, including Basel, Olten, Schaffhausen, Bülach, Zürich discussed before, as well as Bern, in the interior of the country, south of Basel; St. Gallen and Chur towards the east of the country. Table 3 shows, per each station, and for the observed and simulated conditions, the variation of the median arrival delays (in s) between the undisrupted and the disrupted situation, plus sample 95% confidence intervals, for the observed value; the attribution of the delay variation estimated by the simulation to reduced entrance delay or freight rerouting; and the daily amount of trains considered. Confidence intervals of the observations are the largest relatively far from the focal node, resulting in less delay observations (Bern, Chur), as well as at stations with a major change in median delays (Basel, Schaffhausen).

OnTime models well the sign and relative size of the variations, less well the absolute values. All stations connected to Basel enjoy a reduction of delays, decreasing with the distance. For Schaffhausen, the simulations show no variation, while in reality the delay substantially worsened. This mismatch depends on the modelling of the entrance of freight trains in the network, which does not conflict with the arrival delay of trains at Schaffhausen, in the model. The stations connected to Schaffhausen are correctly identified having an increase in delay (see Bülach); in Zürich, both effects interact, with a small net increase of delay. The effects for Schaffhausen and Chur affect much less traffic than busy stations like Basel, Olten, Zürich.

In general, OnTime underestimates by a factor 4 to a factor 6 the magnitude of effects, even though their relative magnitude is in good agreement with observation. One reason for this is the usage of exponential distributions in OnTime, which do not replicate well the long tails that reality showed. Moreover, the passenger traffic at all stations includes many local trains, which (depending on the timetable, and the station) might have a larger or smaller degree of interaction with each other at microscopic level, due to their specific platform used at stations, route chosen in the interlocking area, and precise departure/ arrival time. The delay of long distance traffic (discussed in the previous sections) is therefore diluted into the general picture. Those effects of delay dilution are weaker in Basel, Chur and Bern. Finally, the interaction of special business rules not considered in a purely operative perspective (availability of vehicles, drivers, passenger transfer, etc) has been more complicated than what OnTime has been able to replicate. The exceptional situation was so special that modelling the same system with some minor changes (as OnTime did) might not have been enough to capture the true magnitude of effects. The extra delay observed in real life compared to the simulation gives an idea of the suboptimal planning of freight traffic done in reality, in a hurry, and under strong time pressure, in the area around Schaffhausen, which had moreover to cope with exceptional situations for train drivers, passengers, freight companies, schedulers, dispatchers, as typical in exceptional situations^42,46^.

**Table 3:** Graphical legend for Figure 4, and observed/simulated performance [s].

Station	Observation	Simulation	Of which reduced delay	Of which rerouting freight	Trains/day
Basel	$-45\pm 12$	− 7	− 4	− 3	97
Olten	$-14\pm 6$	− 3	− 1	− 2	114
Bern	$3 \pm 9$	− 7	− 6	− 2	35
Zürich	$4 \pm 4$	1	1	1	171
Chur	$-5 \pm 14$	− 9	− 6	− 1	14
Schaffhausen	$23 \pm 13$	0	0	0	17
Bülach	$25 \pm 7$	5	0	5	62
St.Gallen	$-9 \pm 6$	0	− 2	2	49

We simulate separately the individual effects of the two explicit changes modelled during the disruption, keeping the other as in the baseline, to understand their respective role and impact. The total net effect is the sum of two opposite effects: the improvement in entrance punctuality at Basel has positive effects on the network about twice as strong than the worsening due to rerouting of freight traffic. The two effects interact at few stations, like Zürich and Basel, but have a reduced interaction, adding up mostly linearly.

In Basel, over a daily total amount of around 100 trains, their observed median delay during the disruption is reduced from 91 to 46 s. The simulator estimates the effects of the reduced entrance delay being 58% of this gap, while the effects of rerouting of freight trains thereby releasing infrastructure capacity, decrease the median delay by the remaining 42%. In both this detailed view and the general picture of Table 3, the two effects are quantifiable. This shows how in railway networks both nodes (decreased delay propagation due to smaller entrance delay) and links (increased/decreased delay propagation when more/less traffic is running) are crucial in propagating non-performance. Moreover, the magnitude of those effects is variable, and depending on prevailing conditions: each additional train running in a congested infrastructure has increasingly larger negative effects, which might not be compensated by running a train less elsewhere. In the specific case, the negative effects of freight train rerouting around Schaffhausen are stronger than the positive effects of train rerouting around Basel.

## Discussion

Transport networks are subject to large-scale changes due to sudden disruptions (i.e. hurricanes, earthquakes; infrastructure collapse; terrorist attacks; pandemics or other limitations to mobility). We exploit those changes as they expose mechanisms which are typically co-occurring and mediated by other factors, to understand railway delay propagation dynamics.

Due to the Rastatt railway disruption, the Rhine Alpine corridor from the Netherlands and northern Germany to southern Germany, Switzerland, and Italy, was effectively split into two, and this had a measurable effect on delay propagation on a large part of the Swiss network. By using the delay of passenger trains as a measure of the network performance, over more than a year worth of operational data, at more than ten stations, the shocks in delay propagation experienced, related to the disruption could be separated from the overall spurious variations and noise in operations performance. As a result of management actions, passenger trains were entering in Switzerland from Germany with lower potentially accumulated delay, and freight trains were circulating in reduced number and entering the country at a different boundary point.

This resulted in an overall positive effect of reduced delay, with large heterogeneity in space^14^, by consistently lower delay propagation of all long-distance passenger traffic in a large area around Basel. The area around Schaffhausen experienced increased delays in its passenger traffic, due to the additional freight traffic. The part of the Swiss networks not directly connected with those two boundary points showed no clear and generalized variation of performance. Similar complex effects of functional changes and reorganization within a system due to a shock, resulting in compensatory, or even superior, local behavior ideally match those which have been long postulated, and recently demonstrated in living beings and humans^47,48^.

The sheer scope of the global effects of the Rastatt disruption spanned multiple countries. Even restricted to the Swiss railway network, this disruption proposes a test case much larger than most comparable studies in the literature discussing effects of disruptions, focusing on a city^25^, or the area around a collapsed single bridge^26^. Due to the high variability encountered over this long duration and large geographical extent, typical statistical tests have limited strength in identifying the difference in delay propagation, and isolate the contribution of the disruption. Newly designed statistical tests were applied to delay analysis in railway networks, performed at different percentile levels, over multiple time scales, and two specific factors (reduced entrance delay for passenger trains and rerouted freight trains). They could significantly differentiate the variations in passenger train delays from the noise. Their respective magnitude has been quantified by simulation, with overall good agreement. The system-wide delay propagation dynamic has been described, with a reduction of delays from Basel propagating along the network during the period, with smaller and less identifiable effects farther away, where the magnitude of other spurious effects becomes relatively larger. For high percentile values (i.e. stronger non-performance) the reduction in delays propagation was larger than for low percentile values, meaning particularly the strongly delayed trains performed better in the disruption period, with an overall stabilizing effect of improved performance.

The variability of delays in real-life operations is extremely high; no model of first order or second order, or with a time series analysis could explain all the observed variance of the data. The simulation model used could replicate some effects with a high degree, especially at network scale, but a lot of improvised and non standard business rules in the aftermath of a disruption challenge the mathematical power of such models at the level of precise nodes. Compared to the state of the art, this work proves that railway disruptions behave distinctively over time, space and processes.

Delay propagation in railways, differently from other networks, happens at microscopic level along links and nodes, through conflicts for infrastructure capacity at block section level; global effects are instead appreciated only at macroscopic scale^35,36,49^. Further empirical studies on other railway networks, or other transport networks can identify to which extent the identified dynamics on nodes and links occur in other networks. The specific mix of discrete availability of links and nodes, and continuous movements makes the study of railway systems particularly interesting. Our study provides evidence supporting the validity of railway delay propagation theories at a network level. In the analysis proposed, we identified how railway networks suffer from local instabilities (i.e. delay propagation is common) even though having global stability (i.e. delay propagation is of limited magnitude, and can be absorbed by buffer times, sufficiently away from the disruption)^50^. Even a highly sophisticated simulator routinely used in practice is not able to replicate all magnitudes of delay variation observed, probably due to the choice of underlying probability distributions, and the limited account to specific routes and service stopping pattern running in a real life network. Topological studies have limited power to describe real-life microscopic delay propagation, and relation traffic-performance. Exponential scaling models or epidemic spreading models could be further extended to include the observed performance-depending vulnerability effects. Simulation^51,52^ or optimization models^53^ need to bridge the large gap between detail and complexity of microscopic and macroscopic studies, which we only scratched in this study^54^.

We focused on operational performance only, during the disruption, and did not aim to quantify economic or social costs of the disruption^23^, rerouting or mode choice of passengers^25,26^, nor to specify mitigation or management actions reducing delay propagation, or disruption impact^17,19^. Further studies should consider impacts on different stakeholders with different performance measures^41^. Agent-based models could help in computing passenger costs, assuming that sufficiently accurate modelling of the non-equilibrium^11^ behavior of passengers during disturbed or disrupted operations can be implemented^18,55^.

Moreover, every disruption is one-of-a-kind, and its occurrence triggers often previously unseen dynamics^25,26^. In the Rastatt disruption, freight traffic experienced very large variations from its planned and usual performance; most probably, passenger demand has also been affected and reduced. It is almost impossible to identify or quantify all those effects, and the reasons why some mitigation actions have been chosen, their objective, and effectiveness^42,56,57^.

The availability of smart decision support^17,19^ is very relevant for design of proactive railway traffic control^19^, and future contingency schemes against disruptions^8,58^. We quantified that the system-wide effect of specific management actions combine linearly, i.e. (higher-order) interaction of delay propagation by management actions is marginal. This allows decentralized, decomposed approaches each optimizing a specific item to solve parts of the problem with limited interaction. Our study indicates how the relation between train volume and performance is non linear, with an increasing vulnerability for each extra train running in a congested network. The benefits of relieving a corridor from some traffic are to be traded off with the increased traffic somewhere else, when proposing rerouting as a railway traffic management action^59,60^.

Finally, one important question is how to manage disruptions of this size, i.e. which optimization model can deal with such extreme conditions; and how to include the newly discovered patterns of delay propagation in timetable design^61,62^. Specifically, it has been observed that a shorter circulation of international trains (travelling between Germany and Switzerland) contributed to substantially better delay performance in an entire region. How to integrate this finding in timetable design, and balance punctuality against the comfort of the passengers that would need to transfer between two more punctual trains?

## Methods

### Non parametric identification of shocks in time series

A disruption is assumed to lead to a significant change in a very short term to the time series (i.e. a fast shock), which remains for a certain amount of time corresponding to the entire disruption duration (persistent degradation), and a significant opposite change at its end (i.e. a second shock recovering the first, while returning to full functionality). The pattern of railway delays is generally highly variable; any difference between any day in the disrupted period and any day in the non-disrupted period can also be imputed to many other sources co-occurring. The goal is to quantify the probability that a difference between two consecutive samples in a time series is a spurious product of existing noises, or is related to a fast, persistent, recovered phenomena, happening at the same dates as the disruption. In this latter case, we assume the variation observed is imputable to the disruption. We define three statistical indicators, at different time scales of 1 week, 1 month, 1 year, to identify in time series a fast persistent and recovered disruption as a shock, stabilization to a disrupted level, and return to normality.

To avoid biases from the variations of the timetable within the day (peak hours, nights) and week (reduced service at weekends), we focus on percentile levels ($p\in P=\{20, 40, 60, 80\}$) of the time series of observed arrival delays collected across seven consecutive days. We ignore extreme values (min, max) as those might be traced to vehicle failures and result in cancelled services, with no relation to the disruption.

For any percentile level $p \in P$ and any day $i \in I$, let $d_{i,p}$ be the difference between the *p*-th percentile of the 7 days right before day *i*, and the 7 days right after day *i*. The distribution $D_{p}$ describes the probability of the difference between the percentile at weekly scope (*D* refers to the set of all $D_{p}$s). Specifically in this distribution $D_{p}$, relating to the beginning/end of the disruption *b*/*e* (respectively), $d_{b,p}$ is the difference between the *p*-th percentile delays of the 7 days before the disruption and the respective value of the first 7 days in the disrupted period; $d_{e,p}$ is analogously the difference between the *p*-th percentile delays of the 7 days before the end of the disrupted period with the mean value of the 7 days after the disruption end. The hypothesis to test is whether those differences are significant, i.e. if $d_{b,p}$ and $d_{e,p}$ are common values in the distribution $D_{p}$, the disruption did not have significant effects; if those two values are extreme events, the disruption had a special impact.

Two metrics $I_{diff}$ and $I_{sum}$ respectively describe how likely (based on the observed samples) the differences computed for the beginning and end shock are to be found in the overall distribution of $D_p$ throughout the entire time series. Formally,

$$\begin{aligned} I_{diff,p}&= P[min(|d_{b,p}|,|d_{e,p} |)>min (|d_{j,p}) |,|d_{k,p}|)], \forall j, k \in I \\ I_{sum,p}&=P[(|d_{b,p}|+|d_{e,p} |)> (|d_{j,p}|+|d_{k,p}|)], \forall j, k \in I \end{aligned}$$

Each $I_{diff,p}$ determines the likelihood that any two periods of two consecutive weeks have a minimum variation in delay, at the percentile level *p*, as large as the minimum between the one observed at the beginning and end of the disruption. Each $I_{sum,p}$ determines the likelihood that any two consecutive periods of 1 week have a cumulative absolute variation in delay, at the percentile level *p*, as large as the cumulative absolute variation in delay observed at begin and end of the disruption. Both metrics can take values between 0 and 1. The higher the value is, the more infrequent the change observed at the date of begin/end of the disruption is, in terms of absolute variation ($I_{diff,p}$) or cumulative shock and recovery ($I_{sum,p}$). The usage of percentiles enables understanding if the small delays (low *p*), or the large delays (high *p*), observed during the disruption are more unlikely. Both metrics are based on the ideas of a rank Wilcoxon test.

We consider the time series analysis for the first stop only after a reference point, as timetables have built-in time buffer times^32,61^ to absorb small delays: an arrival delay does not result in a departure delay. Buffer times in timetables are specific to public transport and railway systems, not observed in topological studies; private modes; and also not in airline or maritime networks in the same extent, as trains have multiple stops closely spaced. By suitable choice of buffer times in a timetable, delays can disappear or magnify over time, and specific stations might have smaller (respectively larger) delays without any specific event as cause^52^. The effect of buffer times can be approximated as a systematic baseline of delay; plus a non-linear noise, which affects and reduces delay differently for punctual and non-punctual traffic. To avoid considering effects of buffer times and timetable design, only services connecting two stations without any intermediate stop in between are considered in the delay comparison. In other words, only the variation of delays (to remove systematic baseline), at a station served by a service immediately afterwards a focal point (to remove non-linear delay reduction due to buffer time) is studied. Due to the different service levels (long-distance Intercity traffic *IC*, Interregional *IR*, regional *RE*, neglecting urban railways), with less/more frequent stops of main/secondary category, the effect at various distances can be estimated (see Tables 1, 2).

A second test looks at longer periods, to identify persistent effects of a shock. This test ignores small fast variations which might be considered shocks by the indicators $I_{diff}$ and $I_{sum}$, but were spurious, such as small holiday periods, or adjusted timetable in case of short events (maintenance, concerts, etc). As the disruption lasted more than 2 months, a time length of 4 weeks is considered, before and after the disruption, to determine a baseline for the hypothetical delay distribution (baseline set) during the disruption period, had the disruption not happened. The delay distribution during the 4 weeks immediately after the beginning of the disruption, and the 4 weeks immediately before the disruption end is taken as description of delay distribution during the disruption (disrupted set). These two distributions are compared by a two-sample Kolmogorov–Smirnov (KS) test, aiming to reject the Null Hypothesis $H_0$, that any two samples in the baseline and disrupted set, come from the same distribution. When needed, we refer to a significance level $\alpha = 0.01$. This test is repeated as $KS_p$ for all given percentile levels *p* considered at daily aggregation, as above.

A third test looks at even longer time horizon, comparing the observed behavior during the disruption with synthetic data that describes the best estimate of how the system might have looked, over an entire year, had the disruption not taken place^25^. The delays, at given percentile levels *p*, are modelled as an ARIMA model, with the parameters yielding the highest AIC; this is trained on the entire dataset of 2017, while excluding the disruption period. For the disruption period, the delays at any given percentile level are computed by a Kalman smoothing on the state space representation of the ARIMA model for filling gaps in time series^63^. This gives a synthetic baseline of the expected value of the percentile levels. We compare the synthetic baseline in the disruption period with the real observed values. Under the assumption of equal variances, a *t*-test aims to reject the Null Hypothesis $H_0$, that there is no difference between the delay of the baseline time series and the observed time series. We repeat this *t*-test for all given percentile levels *p* considered at daily aggregation. When needed, we use a significance level $\alpha = 0.01$.

Finally, an absolute metric of the deviation magnitude (measured in seconds) is reported as the Mean Quantile Deviation (MQD), for all percentile levels *p*, between the observed data during the disruption, and the synthetically generated baseline data used also in the *t*-test. A positive deviation is an increase in the delay during the disruption; a negative deviation is a decrease in the delay during the disruption.

### Stochastic railway operations simulation

We use the mesoscopic stochastic railway simulator OnTime, which is based on large-scale Monte-Carlo analysis of probability distributions for trains departures, running time, stopping time at platforms, as well as for infrastructure-constrained train interactions, transfer connections between trains, possibly track works, and secondary delays. Input data include a timetable structure and perturbations in input (primary delays, considering all implicit sources). Based on the planned timetable, and business rules including priorities, primary delays are propagated to the running traffic, determining output propagated delay per each train and station. OnTime considers all traffic running, including passenger and freight traffic, at mesoscopic level, that is ignoring specific signals, but modelling multiple block sections along the lines, and modelling stops. Such a model is much closer to the actual domain processes than general models proposed elsewhere^8,31,36^.

All delay probabilities are assumed expressed as a combination of a negative exponential distribution, plus an additional Dirac distribution for punctual trains. In other terms, delays are compactly represented by two parameters, an average delay (including the variance and expected value of the positive delays), and an intensity (i.e. how many events are actually delayed, and how many are not delayed)^39^. We used a calibrated OnTime model based on the official timetable 2017, and primary delays replicating reality, as provided by the Swiss railways SBB^64^. The possibilities to use different distributions (for instance, Weibull as in^65^) is left to future research, when a simulator and related calibrated parameters would be available. OnTime considers the entire daily timetable over 24 h, and both passenger and freight traffic, respectively 9181 passenger trains (1126 long-distance trains, 8055 regional trains), plus 1150 freight trains. The model has been further calibrated to match the baseline (1 week before the disruption) and disrupted (during the disruption, when the amount and route of freight traffic entering Switzerland stabilized), by adjusting the number of freight trains running, their entrance point in the network, and the input punctuality of passenger trains entering Switzerland from Germany. For both cases, the figure of merit of the calibration was the quantile absolute deviation (QAD) between the measured entrance delay in the Swiss network (upstream of Basel SBB) and the one considered in OnTime. We used affine variations for the parameters of the delay distributions (same values for all affected trains), optimized by a line search.

The entrance delay of passenger trains has been changed, its volume kept constant. The number of freight trains was changed as in the observed data, over a total of 116 areas of the national railway network. Additional freight trains are considered in the corridor Schaffhausen–Zürich–Gotthard, and fewer freight trains are considered in the corridor Basel–Zürich–Gotthard. In this latter, we decreased the train-path usage of freight, i.e. the trains are stochastically running with a lower probability; such reduction ranges between 58 and 80% for the affected areas in the network. The calibration of parameters at network level is correct within 0.1% punctuality (observed 93.08% vs simulated 92.93%).

## Supplementary information

Supplementary Information.
