# Author Correction: Empirical dynamics of railway delay propagation identified during the large-scale Rastatt disruption

**DOI:** 10.1038/s41598-021-83830-9

**Published:** 2021-02-16

**Authors:** Beda Büchel, Thomas Spanninger, Francesco Corman

**Affiliations:** Institute for Transport Planning and Systems, ETH Zürich, 8093 Zürich, Switzerland

Correction to: *Scientific Reports* https://doi.org/10.1038/s41598-020-75538-z, published online 29 October 2020

The Supplementary Information file that accompanies this Article contains errors, where the numbering of tables and references is incorrect. Additionally, the reference list in the Supplementary Information file is omitted.

The corrected Supplementary Information file is linked to this correction notice.

## Supplementary Information

Supplementary Information.

