# Supplementary material for: Author Correction: Empirical dynamics of railway delay propagation identified during the large-scale Rastatt disruption
Source: Sci Rep. 2021 Feb 16;11:4308. doi: 10.1038/s41598-021-83830-9 (PMC7887225; doi:10.1038/s41598-021-83830-9)
Supplement: Supplementary file 1 — Supplementary Information. [file 41598_2021_83830_MOESM1_ESM.pdf]

Supplementary material - Empirical dynamics of railway delay propagation identified during the large-scale Rastatt disruption

Beda Büchel[1], Thomas Spanninger[1], and Francesco Corman[1,*]

[1]ETH Zürich, Institute for Transport Planning and Systems, Zürich, 8093 Switzerland
[*]corman@ethz.ch

ABSTRACT

Transport networks are becoming increasingly large and interconnected. This interconnectivity is a key enabler of accessibility; on the other hand, it results in vulnerability, i.e. reduced performance, in case any specific part is subject to disruptions.
We analyse how railway systems are vulnerable to delay, and how delays propagate in railway networks, studying real-life delay propagation phenomena on empirical data, determining real-life impact and delay propagation for the uncommon case of railway disruptions. We take a unique approach by looking at the same system, in two different operating conditions, to disentangle processes and dynamics that are normally present and co-occurring in railway operations. We exploit the unique chance to observe a systematic change in railway operations conditions, without a correspondent system change of infrastructure or timetable, coming from the occurrence of the large-scale disruption at Rastatt, Germany, in 2017.
We define new statistical methods able to detect weak signals in the noisy dataset of recorded punctuality for passenger traffic in Switzerland, in the disrupted and undisrupted state, along a period of one year. We determine how delay propagation changed, and quantify the heterogeneous, large-scale cascading effects of the Rastatt disruption towards the Swiss network, hundreds of kilometers away.
Operational measures of transport performance (i.e. punctuality and delays), while globally being very decreased, had a statistically relevant positive increase (though very geographically heterogeneous) on the Swiss passenger traffic during the disruption period. We identify two factors for this: (1) the reduced delay propagation at an international scale; and (2) to a minor extent, rerouted railway freight traffic; which show to combine linearly in the observed outcomes.

This document to be used only as supplementary information

version of February 3, 2021

Supplementary information

The 2017 railway disruption at Rastatt

On 12 August 2017, a track settlement occurred between Baden-Baden and Rastatt (Germany) due to the construction of a new railway tunnel. The affected area is part of the 2800 km long Rhine-Alpine Corridor, the largest transport corridor by volume in Europe, stretching from the north sea ports (Rotterdam) to Italy (Genoa). During the disruption, the German railway operator Deutsche Bahn (DB) had to take out of service a track section of around 20 kilometers, and cancel or adjust the service of the trains accordingly. The normal operations resumed on 2 October 2017. Such a disruption has been one of the economically most relevant disruption in the decade. The quantifiable costs in industrial and manufacturing terms amount to more than 2 billion euro. From those, about 12 million euros per week are related to freight companies' losses, according to the European Railways Network[1,2]. Diversions had to be put in place for the 200 freight trains, from different operators, that travel every day on the Rhine Alpine corridor. Overall, it has been estimated that most freight trains were able to run, via a set of very complex diversion routes, as the most direct ones were affect by maintenance works, or with different power systems, thus sometimes requiring diesel locomotives[3] or driving authorization for train drivers. The monitoring of freight at the alpine crossing (Gotthard and Lötschberg tunnels) estimates about 1500 trains being cancelled, and 400 being rerouted. Other statistics would suggest that two thirds of the expected volume of freight traffic was actually running on the alpine crossing[4]. The precise estimation is of course difficult as freight trains took diversions which might have not been properly recorded; and some freight trains were not directed towards the other side of the Alps, thus not being completely traceable by the current monitoring of the alpine crossings[5].

Also the passenger transport was affected. In fact, it is difficult to quantify the direct compensation costs for passengers, extra money required for running replacement services, etc. For instance, some train vehicles were unable to reach their maintenance workshop, which was on the other side of the disruption, thus leading to additional cascading effects of service cancellation[3]. Even harder is to estimate the economic losses from modal shift, possible transfers, and extra bus services, leading to crowding and time lost for passengers (up to two hours extra travel time have been recorded). To organize the replacement services for about 30000 passengers, 450 shuttle buses runs have been organized per day, across the main stations[6]. The main effects resulted in locally cancelled trains, even though the global circulation of trains was maintained. For instance, all trains scheduled to run between Germany and Switzerland were in fact running in Switzerland, but being possibly short turned somewhere (in Offenburg) before reaching the disruption, and not reaching the originally planned destination (Hamburg or Dusseldorf) on the opposite side of the disruption. This management action has been put into place quickly after the disruption and remained relatively stable. Concerning freight traffic, the management actions have been evolving over time[4]. In the first days, most freight companies were just waiting for a quick resolution, but when the scale of the disruption become evident, some way of keeping the freight traffic running had to be found. Some freight trains have been cancelled, some have been globally rerouted between their origin and their destination, for instance not crossing Switzerland, or entering Switzerland from the entry point at Schaffhausen and not in Basel SBB as planned.

Numerical evaluation of the effects around Lausanne

The station of Lausanne is similar to Basel for amount of traffic and passengers, and geographical position (close to the French border). No variation is assumed to have occurred there. The delays of passenger trains with a direct connection from Lausanne are analysed in Table 1 by the metrics proposed at neighboring stations (Morges, Vevey, Fribourg, Yverdon-les-Bains).

Data used

All our analysis is based on recorded train operations (i.e. deviations between arrival/departure time recorded at planned stops, and the planned times). We focus only on the effects visible in the Swiss railway network. We base all our studies on open data for the years 2017, as the timetable changed at the end of 2017, thus prohibiting a direct specific comparison of recorded operations throughout the two years. The timetable year 2017 started on 11 December 2016 and ended on 12 December 2017. It is known that during the summer of 2017 multiple construction works in southern Germany have been planned and executed, with some effects on transport performance in Germany and Switzerland. In Figure 2, we included also timetable year 2018 (starting 13 December 2017 and ending 8 December 2018) for graphical purposes.

A snapshot of the data (available at https://opentransportdata.swiss/en/dataset/istdaten) is reported in Table 2.

Some more detail about the stations investigated is reported in Table 3.

Indicator	p	Lausanne	Morges	Vevey	Fribourg	Yverdon
d [min]	-	0	10	15	24	43
I_{diff}	0.8	.	0.573	0.713	0.165	0.669
	0.6	.	0.497	0.124	0.245	0.450
	0.4	.	0.598	0.125	0.608	0.554
	0.2	.	0.359	0.446	0.868	0.128
I_{sum}	0.8	.	0.248	0.586	0.614	0.46
	0.6	.	0.416	0.305	0.619	0.439
	0.4	.	0.342	0.251	0.773	0.518
	0.2	.	0.170	0.341	0.771	0.219
KS-test	0.8	.	0.143	0.226	0.007	0.865
	0.6	.	0.019	0.717	0.124	0.589
	0.4	.	0.017	0.824	0.069	0.675
	0.2	.	0.243	0.919	0.105	0.759
t-test	0.8	.	0.016	$6.0\ 10^{-4}$	$1.0\ 10^{-4}$	0.791
	0.6	.	$9.5\ 10^{-3}$	$5.0\ 10^{-4}$	$3.1\ 10^{-3}$	0.659
	0.4	.	0.066	$1.4\ 10^{-4}$	$1.1\ 10^{-3}$	0.720
	0.2	.	0.923	$< 10^{-05}$	$2.0\ 10^{-3}$	0.392
MQD	0.8	.	-23.45	-29.11	-39.75	+3.50
	0.6	.	-17.30	-14.49	-19.04	+4.59
	0.4	.	-9.11	-9.83	-12.53	+3.26
	0.2	.	-0.31	-7.29	-8.03	+7.05

Table 1. Metrics for the Lausanne area.

BETRIEBSTAG	FAHRT BEZEICHNER	BETREIBER ID	BETREIBER ABK	BETREIBER NAME	PRODUKT ID
26.03.2020	85:11:17363:001	85:11:00	SBB	Schweizerische Bundesbahnen SBB	Zug
26.03.2020	85:11:17363:001	85:11:00	SBB	Schweizerische Bundesbahnen SBB	Zug
26.03.2020	85:11:17363:001	85:11:00	SBB	Schweizerische Bundesbahnen SBB	Zug
26.03.2020	85:11:964:001	85:11:00	SBB	Schweizerische Bundesbahnen SBB	Zug

LINIEN ID	LINIEN TEXT	UMLAUF ID	VERKEHRS MITTEL TEXT	ZUSATZ FAHRT TF	FAELLT AUS TF	BPUIC	HALTE STELLEN NAME
17363	S3		S	FALSE	FALSE	8500010	Basel SBB
17363	S3		S	FALSE	FALSE	8500023	Liestal
17363	S3		S	FALSE	FALSE	8500218	Olten
964	IC61		IC	FALSE	FALSE	8500218	Olten

ANKUNFTS ZEIT	AN PROGNOSE	AN PROGNOSE STATUS	ABFAHRTS ZEIT	AB PROGNOSE	AB PROGNOSE STATUS	DURCH FAHRT TF
26.03.2020 17:23	26.03.2020 17:22:02	REAL	26.03.2020 17:31	26.03.2020 17:31:56	REAL	FALSE
26.03.2020 17:47	26.03.2020 17:46:27	REAL	26.03.2020 17:47	26.03.2020 17:47:25	REAL	FALSE
26.03.2020 18:10	26.03.2020 18:09:16	REAL			PROGNOSE	FALSE
26.03.2020 10:30	26.03.2020 10:31:11	REAL	26.03.2020 10:33	26.03.2020 10:35:45	REAL	FALSE

Table 2. Example data of the operational data used. The difference between column ANKUNFTS_ZEIT and AN_PROGNOSE determines the arrival delay: in this case respectively (-58 sec, -33 sec, -44 sec, +71 sec).

Focus point	Stop	Stop category	Considered trains	Distance	Trains/hour
Basel SBB	Basel SBB	Boundary	TGV, ICE, IC	-	2.5
	Olten	Main	TGV, ICE, IC	24 min	1.5
	Zürich	Main	TGV, ICE, IC	53 min	2
	Liestal	Secondary	ICE, EC, IC, IR	9 min	2.5
	Rheinfelden	Secondary	IR	12 min	2
Schaffhausen	Schaffhausen	Boundary	IC, IR, RE	-	0.5
	Zürich	Main	IC, IR	36 min	1
	Bülach	Secondary	RE	19 min	1
Lausanne	Lausanne	Benchmark	IC, IR	-	-
	Fribourg / Freiburg	Main	IC	24 min	1.5
	Yverdon-les-Bains	Main	IC	43 min	1
	Morges	Secondary	IR	10 min	2
	Vevey	Secondary	IR	13 min	2

Table 3. The investigated passenger trains running between focus points and investigated stations. In decreasing order of service level: TGV, ICE, EC are different international long-distance trains; IC is intercity traffic; IR is interregional traffic, RE is regional traffic.

References

1. European Rail Freight Association ERFA. *Study finds Rastatt incident to have caused losses of more than 2 billion* (2018). http://erfarail.eu/news/the-economic-impact-of-rastatt.
2. BLS cargo. The unspoken costs of rail disruptions: the consequences of Rastatt on the economy and customer confidence. Talk at general assembly ERFA (2018).
3. Deutsche Bahn Group. *Integrated Report* (2017).
4. UVEK Eidgenössisches Departement für Umwelt and Verkehr, Energie und Kommunikation. *Verlagerungsbericht 2017* (Bern, 2018). https://www.bav.admin.ch/bav/de/home/themen-a-z/verlagerung/verlagerungsbericht.html.
5. Hanseatic Transport Consultancy HTC. *Volkswirtschaftliche Schäen aus dem Rastatt-Unterbruch-Folgenabschützung für die schienenbasierte Supply-Chain entlang des Rhine-Alpine Corridor* (European Rail Freight Association ERFA, 2018). http://erfarail.eu/uploads/2018_April%20Studie-1524476846.pdf.
6. Deutsche Bahn Inside. *Rheintalbahn: Sperrung aufgehoben Verkehr rollt wieder* (2017).
